# Infective Endocarditis Caused by Carbapenemase-Producing Klebsiella pneumoniae on a Prosthetic Valve: A Case Report and Review of the Literature

**DOI:** 10.7759/cureus.64639

**Published:** 2024-07-16

**Authors:** Sanae Azelmat, Tarik Baadi, Elmostafa Benaissa, Abdelhamid Jaafari, Mariama Chadli

**Affiliations:** 1 Microbiology, Mohammed V Military Training Hospital, Mohammed V University, Rabat, MAR; 2 Anesthesia and Intensive Care, Mohammed V Military Training Hospital, Mohammed V University, Rabat, MAR; 3 Bacteriology, Mohammed V Military Training Hospital, Rabat, MAR

**Keywords:** carbapenemase-producing klebsiella pneumoniae, intensive care unit, vegetation, prosthetic valve, infective endocarditis

## Abstract

Infective endocarditis (IE) is a rare but severe disease due to frequent and serious complications. Healthcare-associated cases often involve *Staphylococcus aureus*, while Gram-negative bacteria such as *Klebsiella pneumoniae*, though rare, pose severe challenges due to their resistance profiles. We report a case of a 68-year-old woman with a history of hypertension and mitral valve replacement 12 years ago, who was admitted to the intensive care unit (ICU) for management of non-traumatic, afebrile altered mental status due to intracerebral hemorrhage from anticoagulant overdose. His stay in the ICU revealed septic shock with multi-organ failure caused by carbapenemase (New Delhi metallo-β-lactamase (NDM))-producing *K. pneumoniae* complicated by IE on the prosthetic mitral valve. Despite treatment with meropenem, colistin, and tigecycline, the patient succumbed to septic shock after 15 days of therapy. This case highlights the importance of close surveillance of nosocomial infections and the need for prompt management strategies integrating medical and surgical approaches to reduce the high mortality associated with such infections.

## Introduction

Infective endocarditis (IE) is a rare disease, estimated at between three and nine cases per 100,000 people per year. It is also a serious disease, with an in-hospital mortality rate of around 20%, due to frequent and severe complications (heart failure and septic complications) [1].

IE on prosthetic valves (PVE) currently accounts for 10-30% of IE cases, with an incidence of 0.5-1% patient-years [2-4]. It is associated with a high in-hospital mortality rate of between 20% and 60% [3,5]. It is considered late-onset if it occurs more than 12 months after surgery, as in our case.

We report a rare and potentially severe case of IE caused by carbapenemase (New Delhi metallo-β-lactamase (NDM))-producing* Klebsiella pneumoniae* on a prosthetic valve, highlighting the specific challenges encountered in the management of such infections.

## Case presentation

This is a 68-year-old woman, with a history of hypertension on angiotensin receptor blockers (ARBs) and mitral valve replacement 12 years ago on vitamin-k antagonists (VKAs). The patient was admitted to the intensive care unit (ICU) for management of non-traumatic, afebrile altered mental status due to intracerebral hemorrhage following accidental overdose of oral anticoagulants. The history of the disease dates back to 14 days before her admission, with the sudden onset of headaches complicated by altered mental status, prompting her consultation in the emergency department, where a brain computed tomography scan was performed, revealing a right cerebellar hematoma and a meningeal hemorrhage. Clinical examination on admission revealed a hemodynamically and respiratorily stable patient, with a Glasgow Coma Scale (GCS) score of 9 attributed to verbal response.

The initial laboratory findings showed a CRP of 73 mg/L, hemoglobin of 11.8 g/dL, platelet count of 238,000/mm³, prothrombin ratio (PR) of 16%, and international normalized ratio (INR) > 4, prompting the decision to antagonize with 10 mg vitamin K + 10 units of fresh frozen plasma (FFP). Since then, the patient's GCS has deteriorated to 6, prompting intubation on neurological grounds, with a control hemoglobin of 9.7 g/dL, PR of 66%, and INR of 1.4, leading to surgical admission for external ventricular drain and transfer to the ICU 24 hours after the procedure. His stay in the ICU was complicated by septic shock with hematological failure related to macrophage activation syndrome, renal failure (kidney disease: improving global outcomes (KDIGO) stage 2 acute kidney injury), hemodynamic failure with increased noradrenaline and adrenaline requirements, and hepatic failure with signs of clinical and biological cholestasis.

**Figure 1:**
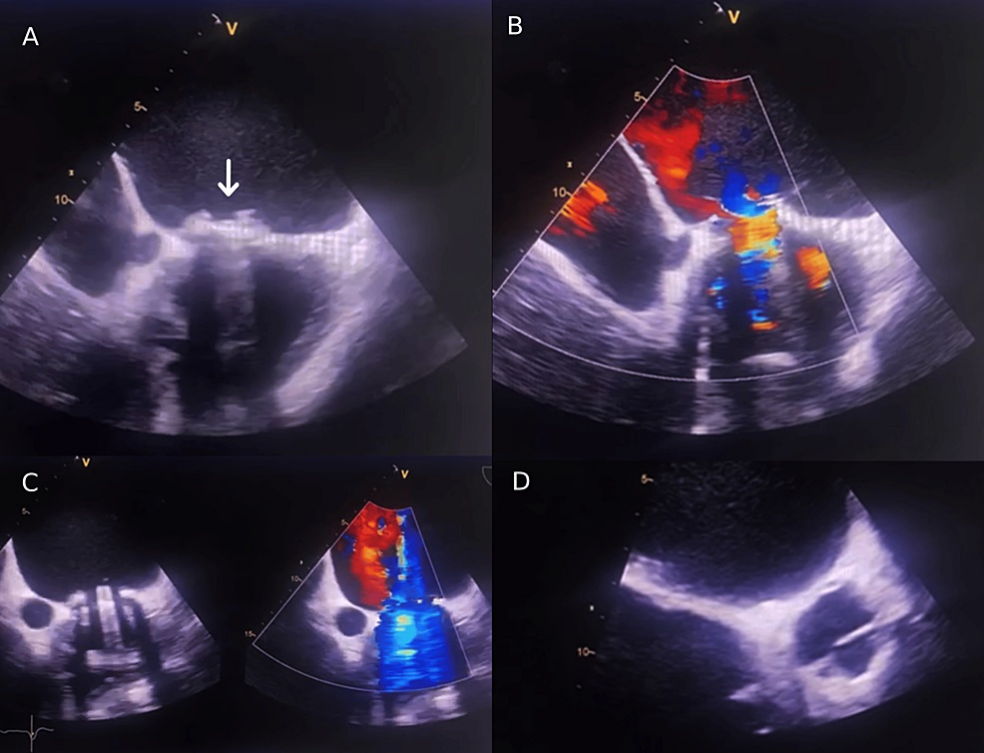
Transesophageal echocardiography showing vegetations on a prosthetic mitral valve (A) with significant periprosthetic leakage (B, C, and D)

Septically, the patient was afebrile but reported purulent secretions at the endotracheal tube. A protected distal sample (PDP) was collected and sent to the bacteriology laboratory. The culture of the PDP revealed a multidrug-resistant *K. pneumoniae* strain at a non-significant threshold suggestive of colonization, according to the significance thresholds proposed by the medical microbiology guidelines (REMIC 2022). The patient has not taken antibiotics. Four days later, the inflammatory parameters (CRP and neutrophil count) were rising. The patient developed a peak fever at 39°C, and a systolic murmur of mitral insufficiency regurgitation was perceived on cardiac auscultation. Therefore, a transesophageal echocardiography (TEE) was performed, revealing vegetations on the prosthetic mitral valve with significant paraprosthetic leakage (Figure 1).

A second PDP was performed due to persistent purulent secretions. Additionally, six sets of blood cultures (aerobic and anaerobic), including three sets from the peripheral venous puncture, were collected at 30-minute intervals and incubated at 37°C in the BD BACTEC™ FX200 system (Becton, Dickinson and Company, Franklin Lakes, NY). The PDP culture was positive, revealing monomorphic colonies at a significant threshold of 10^5^ CFU/mL, identified as multi-drug resistant *K. pneumoniae* based on antibiotic susceptibility testing. All three sets of blood cultures were positive, with positivity time ranging from three hours to eight hours. Direct examination with a Gram stain of the different series revealed the presence of Gram-negative bacilli. Broths were plated on enriched medium (blood agar) and ordinary medium (Bromocresol purple agar) and incubated at 37°C. After 24 hours of incubation, the growth of mucoid and confluent colonies was observed on both. The colony identification by mass spectrometry VITEK® MS PRIME MALDI-TOF (BioMérieux, Marcy l’Etoile, France) confirmed *K. pneumoniae*.

The susceptibility testing by agar diffusion method, conducted according to the European Committee on Antimicrobial Susceptibility Testing (EUCAST) 2023 guidelines, objectified a strain resistant to all antibiotics tested by this technique [6]. This resistance profile was confirmed by a microdilution method using Sensititre™ Gram-negative GNX2F AST Plate (Thermo Fisher Scientific™, Waltham, MA), the results of which are shown in Table 1. Our strain remained sensitive to colistin and tigecycline. A molecular study was conducted using rapid real-time PCR, Xpert® Carba-R PCR assay* *(Cepheid, Sunnyvale, CA), capable of detecting genes encoding the most common carbapenemases, such as KPC, NDM, VIM, IMP, and OXA-48, within 50 minutes. This analysis detected the carbapenemase NDM gene. The diagnosis of IE was established based on the Duke criteria [7].

**Table 1:** Susceptibility results for the Klebsiella pneumoniae isolate MIC, minimum inhibitory concentration

Antibiotics	MIC (mg/L)	Susceptibility
Amikacin	> 32	Resistant
Aztreonam	> 16	Resistant
Cefepime	> 16	Resistant
Cefotaxime	> 32	Resistant
Ciprofloxacin	> 2	Resistant
Colistin	= 1	Sensitive
Doripenem	> 2	Resistant
Doxycycline	> 16	Resistant
Ertapenem	> 4	Resistant
Gentamicin	> 8	Resistant
Imipenem	> 8	Resistant
Levofloxacin	> 8	Resistant
Meropenem	> 8	Resistant
Minocycline	> 16	Resistant
Piperacillin/tazobactam 4	> 64/4	Resistant
Polymixin B	> 4	Resistant
Ticarcillin/clavulanic acid	> 128/2	Resistant
Tigecycline	= 0.25	Sensitive
Tobramycin	> 8	Resistant
Trimetoprim/sulfamethoxazole	> 4/76	Resistant

The patient was treated with meropenem + colistin + tigecycline, initially showing good clinical and biological improvement evidenced by a decrease in biomarkers. However, after 15 days of treatment, the patient passed away due to septic shock accompanied by multi-organ failure.

## Discussion

IE usually follows bacteremia. The normal valvular endothelium resists infection, but its alteration due to valvular disease, whether native or prosthetic, exposes the thrombogenic extracellular matrix of the sub-endothelium and triggers platelet adhesion and activation, followed by rapid colonization by circulating microorganisms. These microorganisms proliferate and form a biofilm, which provides resistance to immune defenses and antibiotic treatments [8].

IE can occur in both community and hospital settings. The pathogens identified in healthcare-associated IE are typically those associated with nosocomial infections, in particular, those of cutaneous origin; *Staphylococcus aureus* is the most frequent bacteria, responsible for 33%-45% of healthcare-associated IE, with no significant difference between nosocomial and non-nosocomial IE. Coagulase-negative *Staphylococcus*, oral streptococci, and enterococci were also among the germs most frequently implicated [9,10].

Other frequent oropharyngeal colonizers, such as the HACEK organisms (*Haemophilus *species, *Aggregatibacter *species, *Cardiobacterium hominis*, *Eikenella corrodens*, and *Kingella *species), which are mainly Gram-negative, contribute to approximately 20% of all cases of endocarditis [11].

In contrast, Gram-negative bacteria, mainly *Enterobacteriaceae*, are rarely incriminated, responsible for 1.8-5.3% of IE cases, 90% of which are of nosocomial origin [2]. Within this group, *K. pneumoniae* is one of the most rarely implicated species, due to its limited ability to adhere to valve endothelial cells compared with the Gram-positive bacteria mentioned above [12].

Nowadays, infections from multidrug-resistant organisms (MDRO) acquired in hospitals are a major worry for clinicians worldwide, as they lead to higher rates of short- and long-term mortality [13]. The rate of MDRO (resistant to three or more specific antimicrobials) causing endocarditis is 23.4% (6.4% of multi-resistant Gram-negative bacteria) based on a multicentric retrospective analysis made in Romania according to the guidelines of the Declaration of Helsinki [11].

The poor prognosis of IE, especially after the development of valvular complications such as large vegetation, abscesses and fistulas, perforations, false aneurysms, and Gerbode defect, leads to perivalvular insufficiency. This complication was found in 7% of mitral mechanical valve cases and highlights the need for prompt management, combining both medical and surgical treatments mainly, to improve survival chances and reduce complications associated with this severe condition [14]. This study holds crucial importance since there have been few cases in the literature of endocarditis caused by carbapenemase-producing *K. pneumoniae* [13,15,16].

## Conclusions

This very rare case of *K. pneumoniae* carbapenemase-producing associated with infective endocarditis on a prosthetic mitral valve highlights the growing threat posed by nosocomial infections caused by Gram-negative bacteria. Antibiotic resistance considerably complicates the management of these infections, requiring an individualized therapeutic approach, often combining different antimicrobial agents and surgical interventions. It highlights the importance of close surveillance of nosocomial infections, implementation of effective preventive measures, and ongoing research into new treatments to tackle these complex clinical challenges.
